# Supplementary material for: Mechanisms of ribosome stalling by SecM at multiple elongation steps
Source: eLife. 2015 Dec 15;4:e09684. doi: 10.7554/eLife.09684 (PMC4737659; doi:10.7554/eLife.09684)
Supplement: Supplementary file 1. — DOI: http://dx.doi.org/10.7554/eLife.09684.030 [file elife-09684-supp1.docx]

**Supplementary file 1. Statistics of data processing and model refinement**

| **Data collection** | |  | |
| --- | --- | --- | --- |
| Electron microscope | | Titan Krios | |
| Voltage (kV) | | 300 | |
| Electron detector | | K2 camera | |
| Defocus range (μm) | | 1-3.5 | |
| Electron dose (e^-^/Å^-2^) | | 16 (frame 2-15) | |
| Pixel size (Å) | | 1.32 | |
|  | **SecM-Gly-RNC** | | **SecM-Pro-RNC** |
| **3D Reconstruction** |  | |  |
| Unit cell (P1) |  | |  |
| a=b=c (Å) | 422.4 | | 422.4 |
| ɑ=β=γ(˚) | 90 | | 90 |
| Particles for final refinement | 60,354 | | 41,501 |
| **Refinement** |  | |  |
| Resolution of unmasked map (Å) | 4.49 | | 4.31 |
| Map sharpening B-factor (Å^2^) | -90.6 | | -62.7 |
| Resolution of masked map (Å)  (50S mask) | 3.6 | | 3.3 |
| R factor† | 0.34 | | 0.35 |
| Fourier Shell Correlation (FSC)* | 0.76 | | 0.79 |
| **Model composition (50S + tRNA + SecM + mRNA)** |  | |  |
| Non-hydrogen atoms | 92,729 | | 94,332 |
| Protein residues | 3,381 | | 3,382 |
| RNA bases | 3,097 | | 3,172 |
| **R.m.s. deviations** |  | |  |
| Bonds length (Å) | 0.0096 | | 0.0104 |
| Bonds angles (˚) | 1.4577 | | 1.4705 |
| **Ramachandran plot** |  | |  |
| Favored (％) | 90.72 | | 90.24 |
| Outliers (％) | 2.50 | | 2.77 |
| **Validation ( proteins)** |  | |  |
| Molprobity score | 3.14 | | 3.09 |
| Good rotamers (%) | 85.10 | | 83.61 |

†R factor = ∑∥F_obs_∣-∥F_calc_∣ ∕ ∑∣F_obs_∣

* FSC = ∑(F_obs_F*_calc_) ∕ (∑∣F_obs_∣^2^ ∑∣F_calc_∣^2^)
